# Prevalence and Factors Associated With Willingness to Sustain Pandemic-Induced Digital Work in the General Population and Moderating Effects of Screen Hours: Cross-Sectional Study

**DOI:** 10.2196/53321

**Published:** 2024-05-28

**Authors:** Jiaying Li, Daniel Yee Tak Fong, Mandy Man Ho, Edmond Pui Hang Choi, Kris Yuet Wan Lok, Jung Jae Lee, WenJie Duan, Janet Yuen Ha Wong, Chia-Chin Lin

**Affiliations:** 1 School of Nursing The University of Hong Kong Hong Kong SAR China (Hong Kong); 2 Department of Social Work East China University of Science and Technology Shanghai China; 3 School of Nursing and Health Studies Hong Kong Metropolitan University Hong Kong SAR China (Hong Kong)

**Keywords:** COVID-19 pandemic, digital work, willingness to sustain, screen time, general population

## Abstract

**Background:**

The pandemic has accelerated digital work transformation, yet little is known about individuals’ willingness to sustain such digital modes and its associated factors. A better understanding of this willingness and its drivers is crucial for guiding the development of future digital work infrastructure, training programs, and strategies to monitor and prevent related health issues.

**Objective:**

This study aims to quantify the general population’s willingness to sustain pandemic-induced digital work, identify its associated factors, and examine how screen time moderates these relationships.

**Methods:**

A cross-sectional study was conducted targeting Hong Kong residents aged ≥18 years who have increased engagement in digital work since the pandemic. Data were collected through self-reported, web-based surveys. Descriptive statistics determined prevalence rates, while structured multiphase logistic regression identified associated factors and explored the moderating effects of screen hour levels.

**Results:**

This unfunded study enrolled 1014 participants from May 2 to June 24, 2022, and completed data analysis within 3 months after data collection. A total of 391 (38.6%; 95% CI 35.6%-41.6%) participants expressed willingness to sustain digital work. Positive factors associated with this willingness included being an employee (odds ratio [OR] 3.12, 95% CI 1.59-6.45; *P*=.001), being health professionals (OR 3.32, 95% CI 1.49-7.82; *P*=.004), longer screen hours (OR 1.09, 95% CI 1.03-1.15; *P*=.002), and higher depression levels (OR 1.20, 95% CI 1.01-1.44; *P*=.04). Conversely, negatively associated factors included older age (OR 0.87, 95% CI 0.81-0.94; *P*=.001), extroversion (OR 0.66, 95% CI 0.51-0.86; *P*=.002), higher eHealth literacy (OR 0.96, 95% CI 0.93-0.98; *P*<.001), perceived greater susceptibility to COVID-19 (OR 0.84, 95% CI 0.74-0.96; *P*=.009), residence in a high-severity COVID-19 community (OR 0.73, 95% CI 0.63-0.84; *P*<.001), having infected individuals in the immediate social circle (OR 0.64, 95% CI 0.46-0.88; *P*=.006), higher BMI (OR 0.94, 95% CI 0.90-0.99; *P*=.02), feelings of being out of control (OR 0.96, 95% CI 0.93-0.98; *P*=.002), and higher fear of COVID-19 (OR 0.96, 95% CI 0.94-0.98; *P*=.001). In addition, a moderating effect of screen hour level (high: >8 h/d; low: ≤8 h/d) influenced the association among 10 factors related to willingness to sustain pandemic-induced digital work, including age, education level, household size, needs for regular medical care, BMI, frequency of both vigorous and moderate physical activities, perceived COVID-19 severity, immediate social circle COVID-19 presence, and fear of COVID-19 (all *P* values for interaction <.05).

**Conclusions:**

The substantial willingness of the general population to sustain digital work after the pandemic highlights the need for robust telework infrastructure, thorough monitoring of adverse health outcomes, and the potential to expand telehealth services among this group. The identification of factors influencing this willingness and the moderating role of screen hours inform the development of personalized strategies to enhance digital work acceptance where needed.

## Introduction

Digital transformation, recognized as one of the most significant benefits of the COVID-19 pandemic, has deeply integrated digital technologies into daily work practices, transforming traditional physical offices into predominantly digital realms [1,2]. Supported by the widespread adoption of videoconferencing and instant messaging platforms, real-time communication has been revolutionized. In addition, learning management systems and project management tools have become indispensable for organizing tasks efficiently and ensuring secure data sharing [3]. This global shift toward digital work environments raises critical questions about their long-term sustainability, as this extent of willingness to sustain such internet-mediated work environments is pivotal not only for shaping future tele-based infrastructure but also for influencing prevention and surveillance of tele-related health outcomes, as well as enhancing our understanding of human acceptance of digital means along with the factors driving this acceptance.

Although extensive research has explored the advantages and drawbacks of digital work environments [4-7], significant gaps remain in understanding how widely individuals are willing to continue in such settings. One notable study revealed that 63.4% of US energy sector employees who transitioned to digital work were reluctant to return to on-site work, preferring to avoid their physical workplaces [8]. Broadening our understanding of this willingness across varied populations is essential for shaping policies on future tele-based infrastructure. This encompasses improvements in remote work features such as cybersecurity and data privacy protections. In addition, these insights are crucial for evaluating the health outcomes associated with the rising trend of telework [6,9], guiding future health recommendations and workplace standards. Furthermore, the positive correlation between telework and telemedicine suggests that this willingness could also influence public health strategies to enhance health care access in increasingly remote settings [10].

Understanding the associated factors of willingness to sustain digital transformations is crucial for guiding strategies to enhance acceptance for needs such as resource conservation, personalized work adjustments, and ensuring adherence to social distancing in future pandemics. To identified associated factors, we used a web-based survey using the Health Belief Model, which effectively predicts health behaviors and encompasses 4 main domains: perceived susceptibility, severity, benefits, and barriers [11]. In this model, knowledge of COVID-19, prior infection history, and eHealth literacy are classified under perceived susceptibility and severity, clarifying how individuals perceive the risks of sustaining digital work. Meanwhile, lifestyle habits and BMI correspond to perceived benefits and barriers, capturing individual evaluations of the pros and cons of sustaining the pandemic-induced digital work. In addition, the Health Belief Model acknowledges the impact of demographic and psychological factors, such as personality traits, on health behaviors, which directly influence both the model’s key domains and health behaviors themselves. Extended screen time in digital work often results in visual strain, musculoskeletal discomfort, and mental fatigue [12,13], which may diminish one’s willingness to continue such arrangements. However, frequent exposure to prolonged screen hours can also enhance proficiency with information and communication technologies [14], thereby improving perceptions of productivity and work-life balance in digital environments. Given these dual effects, the influence of various factors on willingness to sustain digital work might differ significantly among individuals based on their levels of screen time exposure. Thus, we also investigated the potential moderating effect of screen time on the association of the aforementioned factors with the willingness to sustain digital work in order to provide a more nuanced understanding of these relationships.

Therefore, this study addresses research gaps by quantifying the general population’s willingness to sustain digital work, identifying influencing factors, and examining how screen time moderates these relationships. Our hypotheses were as follows: (1) a segment of the population will prefer continued digital work, (2) various factors influence this willingness, and (3) screen time affects this willingness and alters the effects of related factors. Findings were expected to enhance understanding of digital work acceptance and its determinants, guiding the development of tele-based work infrastructure, health outcome monitoring, and tailored strategies to increase willingness where needed.

## Methods

### Study Design

This was a cross-sectional study conducted in Hong Kong using a web-based survey. The methodology of the web-based survey was reported in accordance with the CHERRIES (Checklist for Reporting Results of Internet E-Surveys) checklist [15].

### Setting

The web-based survey was conducted among Chinese adults in Hong Kong from May 2 to June 24, 2022. This period was approximately 2 months after the Omicron surge (from February 28 to March 6, 2022), and social distancing measures were still in place.

### Participants and Sample Size

The web-based survey used convenience sampling to include participants who were Chinese-reading adults aged 18 years or older residing in Hong Kong. We excluded individuals who reported no increased engagement in digital working or learning since the onset of the COVID-19 pandemic via a self-reported question. The sample size was determined using the rule of thumb, that is, a minimum of 10 events per variable [16]. Considering up to 19 variables in the regression model, a total of 190 subjects experiencing the event were required. Based on a prior study in which the event rate of reluctance to return to the physical workplace was 63.4% (211/333) [8], a total of 300 participants would be needed.

### Variables

#### Overview

Multimedia Appendix 1 contains the web-based questionnaire, detailing each variable included in this study.

#### Sociodemographics

Sociodemographic variables included sex, age, marital status, education level, employment status, perceived social rank, whether they were health professionals, needs for regular medical care, number of children younger than 18 years, and household size. In addition, BMI was also recorded.

#### COVID-19–Related Knowledge and Infection

We assessed eHealth literacy, COVID-19–related knowledge, and COVID-19 infection status. eHealth literacy was assessed using the validated Chinese eHealth Literacy Scale [17], which evaluates respondents’ knowledge, comfort, and skills in using electronic health information. The 8-item scale uses a 5-point Likert scale, with higher scores indicating greater eHealth literacy. In addition, COVID-19–related knowledge was measured using 6 items from the World Health Organization’s behavioral survey on COVID-19 [18], focusing on perceived knowledge and severity. Each item was rated on a 7-point Likert scale, with higher scores indicating greater knowledge or severity. Moreover, COVID-19 infection status was assessed with 2 questions asking whether participants or someone in their immediate social circle had contracted COVID-19 [18], with responses as “yes” or “no.”

#### Lifestyle

Self-reported lifestyle variables included alcohol and tobacco use, frequency of vigorous and moderate physical activities, screen time, and sitting hours. Tobacco and alcohol consumption were assessed using an 8-point Likert scale from “never” to “everyday.” The frequency of both vigorous and moderate physical activities was rated on a 6-point Likert scale from “none” to “5 days or more.” Participants reported their average daily screen hours and sitting hours by entering an integer number of hours. For analysis, screen hours were dichotomized into 2 groups: those reporting more than 8 hours were categorized as the “high screen hour group,” and those reporting 8 hours or fewer were categorized as the “low screen hour group.”

#### Perceived Personality

We assessed participants’ personality identities with a self-reported single question—“Do you primarily identify as an introvert or an extrovert?”—as in other studies [19,20].

#### Psychological Status

Psychological outcomes including anxiety, depression, fear of COVID-19, and feelings of being out of control were evaluated. Anxiety and depression were measured using the 4-item Patient Health Questionnaire-4, with both subscales showing a Cronbach α value of 0.80 [21,22]. Responses were rated on a 4-point Likert scale, with higher scores indicating more severe symptoms. In addition, fear of COVID-19 was assessed using an 8-item scale validated in Chinese populations, with a Cronbach α value of 0.93 [22]. Items were scored on a 5-point Likert scale, with higher scores indicating greater fear. Moreover, feeling of being out of control was measured by a 7-item scale, with a Cronbach α value of 0.91 [18], using a 6-point Likert scale, where higher scores indicated a stronger perception of loss of control.

#### Willingness to Sustain Pandemic-Induced Digital Work Mode

We used a single direct question to assess participants’ willingness to return to a prepandemic work mode, with binary response options of “yes” or “no.”

### Data Collection

Between May 2 and June 24, 2022, we collected data from the Hong Kong community by developing an open web-based survey. Prior to the main data collection, we piloted it with 30 people to ensure the usability and functionality of the survey. The survey link was then distributed through email and text messages. To ensure a diverse representation of work environments and statuses, we partnered with an established local survey provider who had access to an extensive participant database. The survey, with 58 questions presented 1 per page, targeted individuals meeting our predefined inclusion criteria. Participation in the survey was voluntary, with no incentives provided. The survey did not use randomized questions or adaptive questioning techniques. However, respondents had the option to revise their responses using a back button, and a completeness check was implemented. To prevent duplicate submissions, we tracked the IP addresses of participants throughout the data collection period. Of the 2398 individuals contacted, 1900 (79.2%) successfully completed the survey, reflecting a notable response rate. Incomplete responses were excluded from data analysis, and no specific time frame was applied to disqualify completed surveys.

### Data Analysis

All statistical analyses were performed using R software (version 4.1.1; R Core Team 2021). After excluding participants who were not currently working, those who reported no changes in their work, or those who did not increase their engagement in digital work since the onset of the COVID-19 pandemic, we used descriptive statistics to summarize the sociodemographic data. Continuous variables that conformed to a normal distribution were summarized using means and SDs, while categorical variables were described by frequency counts.

Associated factors of willingness to sustain digital work were identified using a 2-step structured multiphase logistic regression, which grouped variables into sequential clusters before analysis to avoid overadjustment and consider causal relationships [23]. In the first stage, independent variables were organized into sequential clusters based on their potential causal relationships, as informed by empirical evidence. That is, variables in a cluster can affect the variables in later clusters but not vice versa. Cluster 1 included sociodemographic variables, including age, sex, education level, marital status, employment status, need for regular medical follow-up before the COVID-19 pandemic, whether practicing health professional, number of children younger than 18 years, household size, house size, perceived social rank, and personality. Cluster 2 included COVID-19–related knowledge, infection status, and lifestyle. BMI was grouped into cluster 3, while psychological variables, including out of control, fear of COVID-19, anxiety, and depression, were considered as cluster 4. Cluster-1 variables were considered immutable, not influenced by changes in lifestyle or other variables in later clusters. Similarly, cluster-2 variables may influence the health outcome variables in clusters 3 and 4. Also, cluster 3’s variable, that is, BMI, was a self-reported variable and presumed to be measured before psychological variables and thus may influence cluster-4 variables. We ultimately had 4 clusters. Our clustering is thus informed by existing knowledge, aiming to reflect a logical order of influence rather than implying direct causality from the data of this cross-sectional study. In the second stage, logistic regression analysis was conducted in 4 phases, with each phase adjusting for significant variables identified in the previous phase. In addition, screen hours were categorized into high (>8 h/d) and low (<8 h/d) levels. We analyzed the moderation effect of screen hour level on the association of each factor with the willingness to sustain, adjusting for corresponding factors within the same phase. Model adequacy was verified using the Hosmer-Lemeshow goodness-of-fit test, and multicollinearity was assessed with the variance inflation factor, setting statistical significance at *P*<.05.

### Ethical Considerations

The institutional review board of The University of Hong Kong—the Hospital Authority Hong Kong West Cluster approved this study (approval UW 20-272). All participants provided informed consent, were briefed on their right to withdraw at any time without repercussions, and participated voluntarily. Measures were taken to ensure privacy and confidentiality through the anonymization of data, with no personal identifiers used in the analysis. No compensation was provided to the participants for their involvement in the study.

## Results

### Data Cleaning and Respondents’ Characteristics

Initially, we received 1900 valid responses. After removing respondents who were job seeking (n=86, 4.5%), laid off (n=20, 1.1%), not in workforce (n=213, 11.2%), retired (n=128, 6.7%), and reported no change or no increased engagement in digital work or learning mode since the COVID-19 pandemic (n=439, 23.1%), we were left with 1014 respondents. Of these, 518 (51.1%) were female respondents. Table 1 summarizes the respondents’ other characteristics.

**Table 1:** Participants’ sociodemographics, COVID-19–related knowledge and infection, lifestyles, and psychological status (n=1014).

Variables			Values
**Age group (years), n (%)**			
	18-24	105 (10.4)	
	25-29	118 (11.6)	
	30-34	148 (14.6)	
	35-39	163 (16.1)	
	40-44	136 (13.4)	
	45-49	99 (9.8)	
	50-54	81 (8)	
	55-59	91 (9)	
	60-64	48 (4.7)	
	≥65	25 (2.5)	
**Sex, n (%)**			
	Female	518 (51.1)	
	Male	496 (48.9)	
**Education levels, n (%)**			
	Primary or below	24 (2.4)	
	Secondary	483 (47.6)	
	College	137 (13.5)	
	Associate degree	101 (10)	
	Bachelor’s degree	257 (25.3)	
	Graduate	12 (1.2)	
**Marital status, n (%)**			
	Married or cohabitation or common law	611 (60.3)	
	Separated or divorced or widowed	19 (1.9)	
	Single	384 (37.9)	
**Occupational status, n (%)**			
	Self-employed	37 (3.6)	
	Student	51 (5)	
	Employee	926 (91.3)	
**Needs for regular medical care, n (%)**			
	No	824 (81.3)	
	Yes	190 (18.7)	
**Practicing health professional, n (%)**			
	No	986 (97.2)	
	Yes	28 (2.8)	
**Personality, n (%)**			
	Introverts	457 (45.1)	
	Extroverts	557 (54.9)	
Number of children younger than 18 years, mean (SD)			0.39 (0.68)
Number of people in the household, mean (SD)			3.38 (1.10)
House size (m^2^), mean (SD)			40.38 (14.36)
Perceived social rank (1=lowest to 5=highest), mean (SD)			2.94 (0.70)
BMI, mean (SD)			23.50 (2.82)
eHealth literacy (8-40), mean (SD)			26.85 (6.18)
Knowledge on COVID-19 (1-7), mean (SD)			4.37 (0.92)
Knowledge on preventing the spread of COVID-19 (1-7), mean (SD)			4.39 (0.94)
Perceived adequacy of knowledge about COVID-19 (1-7), mean (SD)			4.38 (0.95)
Perceived susceptibility to an infection with COVID-19 (1-7), mean (SD)			4.28 (1.28)
Perceived severity of contracting COVID-19 (1-7), mean (SD)			4.23 (1.26)
Severity of the spread of COVID-19 in your community (1-7), mean (SD)			4.48 (1.30)
**Ever infected with COVID-19, n (%)**			
	Yes	374 (36.9)	
	No	640 (63.1)	
**Someone in your immediate social circle infected with COVID-19, n (%)**			
	Yes	695 (68.5)	
	No	319 (31.5)	
Lifestyles, mean (SD)			
Frequency of drinking alcohol per week (0=never to 8=everyday), mean (SD)			2.92 (1.70)
Frequency of smoking per week (0=never to 8=everyday), mean (SD)			2.52 (2.65)
Screen hours per day (hours), mean (SD)			6.48 (3.44)
**Screen hour group, n (%)**			
	Low screen hour group (≤8 hours per day)	783 (77.2)	
	High screen hour group (>8 hours per day)	231 (22.8)	
Sitting hours per day (hours), mean (SD)			7.46 (3.37)
Frequency of vigorous physical activities per week (0=none to 6=5 days or more), mean (SD)			1.73 (1.22)
Frequency of moderate physical activities per week (0=none to 6=5 days or more), mean (SD)			1.98 (1.28)
Out of control (7-42), mean (SD)			25.37 (5.92)
Fear of COVID-19 (8-40), mean (SD)			20.12 (7.54)
Anxiety (2-8), mean (SD)			2.91 (1.15)
Depression (2-8), mean (SD)			2.76 (1.13)

### Willingness to Sustain Digital Work

Among the 1014 participants, 391 (38.6%; 95% CI of 35.6%-41.65%) expressed a willingness to sustain digital work.

### Associated Factors of Willingness to Sustain Digital Work

Table 2 presents the results of the structured multiphase logistic regression. The variance inflation factor values for all phases of the regression ranged from 1.02 to 1.52, indicating low correlation among the independent variables. The Hosmer-Lemeshow goodness-of-fit test results for phase 1 (*χ*^2^_8_=3.58; *P*=.89), phase 2 (*χ*^2^_8_=10.85; *P*=.21), phase 3 (*χ*^2^_8_=6.81; *P*=.56), and phase 4 (*χ*^2^_8_=8.17; *P*=.42) all demonstrated adequacy. The statistics for the variables, which function as adjusting variables in the subsequent phase, are shown in Multimedia Appendix 2.

In phase 1, older age (odds ratio [OR], 0.87, 95% CI 0.69-1.10; *P*=.001) and being an extrovert (OR 0.66, 95% CI 0.51-0.86; *P*=.002) were negatively associated with the willingness to sustain, whereas employees (OR 3.12, 95% CI 1.59-6.45; *P*=.001) and health professionals (OR 3.32, 95% CI 1.49-7.82; *P*=.004) showed increased willingness.

In phase 2, higher eHealth literacy (OR 0.96, 95% CI 0.93-0.98; *P*<.001), perceived higher susceptibility to COVID-19 (OR 0.84, 95% CI 0.68-1.04; *P*=.009), living in a high-severity COVID-19 community (OR 0.73, 95% CI 0.63-0.84; *P*<.001), and having people infected in the immediate social circle (OR 0.64, 95% CI 0.46-0.88; *P*=.006) negatively influenced willingness to sustain. Conversely, longer screen hours (OR 1.09, 95% CI 1.03-1.15; *P*=.002) were positively associated willingness to sustain.

**Table 2:** Structured multiphase logistic regression of willingness to sustain digital work (n=1014).

Variables					OR^a^ (95% CI)		*P* value		VIF^b^	
**Phase 1**										
		Age (years; 1-10)^c^			0.87 (0.81-0.94)		<.001		1.35	
		Educational level (1-6)^d^			0.98 (0.88-1.10)		.77		1.16	
		**Marital status (reference: single)**								1.17
			Married or cohabitation or common law	1.24 (0.87-1.78)		.24				
			Separated or divorced or widowed	0.61 (0.16-1.88)		.42				
		**Sex (reference: female)**								1.03
			Male	0.79 (0.61-1.04)		.09				
		**Occupational status (reference: student)**								1.04
			Self-employed	1.48 (0.47-4.42)		.49				
			Employee	3.12 (1.59-6.45)		.001				
		Needs for regular medical care (yes vs no)			0.83 (0.57-1.18)		.30		1.05	
		Practicing health professional (yes vs no)			3.32 (1.49-7.82)		.004		1.02	
		Number of children younger than 18 years			0.87 (0.69-1.10)		.26		1.24	
		Number of people in the household			1.00 (0.87-1.15)		.99		1.17	
		House size, m^2^			1.00 (0.99-1.01)		.46		1.13	
		Perceived social rank			1.02 (0.83-1.25)		.85		1.08	
		Personality (extroverts vs introverts)			0.66 (0.51-0.86)		.002		1.01	
**Phase 2**										
		eHealth literacy			0.96 (0.93-0.98)		<.001		1.08	
		Knowledge on COVID-19			0.84 (0.68-1.04)		.12		1.39	
		Knowledge on preventing the spread of COVID-19			1.11 (0.90-1.37)		.35		1.44	
		Perceived adequacy of knowledge about COVID-19			0.9 (0.73-1.09)		.27		1.34	
		Perceived susceptibility to an infection with COVID-19			0.84 (0.74-0.96)		.009		1.17	
		Perceived severity of contracting COVID-19			1.08 (0.93-1.25)		.32		1.31	
		Severity of the spread COVID-19 in your community			0.73 (0.63-0.84)		<.001		1.30	
		Ever infected with COVID-19 (yes vs no)			1.03 (0.74-1.42)		.88		1.14	
		Someone in your immediate social circle infected with COVID-19 (yes vs no)			0.64 (0.46-0.88)		.006		1.12	
		Frequency of alcohol drinking per week			1.01 (0.92-1.11)		.82		1.13	
		Frequency of smoking per week			0.97 (0.92-1.03)		.36		1.11	
		Screen hours (0-24 hours per day)			1.09 (1.03-1.15)		.002		1.37	
		Sitting hours (0-24 hours per day)			0.97 (0.92-1.02)		.28		1.35	
		Frequency of vigorous physical activities per week			0.95 (0.83-1.08)		.41		1.18	
		Frequency of moderate physical activities per week			1.1 (0.97-1.24)		.15		1.18	
**Phase 3**										
	BMI				0.94 (0.90-0.99)		.02		1.03	
**Phase 4**										
	Out of control				0.96 (0.93-0.98)		.002		1.22	
	Fear of COVID-19				0.96 (0.94-0.98)		.001		1.32	
	Anxiety				1.07 (0.90-1.28)		.44		1.52	
	Depression				1.2 (1.01-1.44)		.04		1.47	

^a^OR: odds ratio.

^b^VIF: variance inflation factor.

^c^Age group: 1-10 corresponding to 18-24, 25-29, 30-34, 35-39, 40-44, 45-49, 50-54, 55-59, 60-64, and older than 65 years, respectively.

^d^Educational level: 1-6 corresponding to primary or below, secondary, college, associated degree, bachelor’s degree, and graduate, respectively.

In phase 3, higher BMI showed negative association (OR 0.94, 95% CI 0.90-0.99; *P*=.02). In phase 4, higher out of control (OR 0.96, 95% CI 0.93-0.98; *P*=.002) and higher fear of COVID-19 (OR 0.96, 95% CI 0.94-0.98; *P*=.001) were negatively associated with willingness to sustain, while higher depression was a risk factor (OR 1.20, 95% CI 1.01-1.44; *P*=.04).

### Moderating Effects of Screen Hour Levels

**Figure 1:**
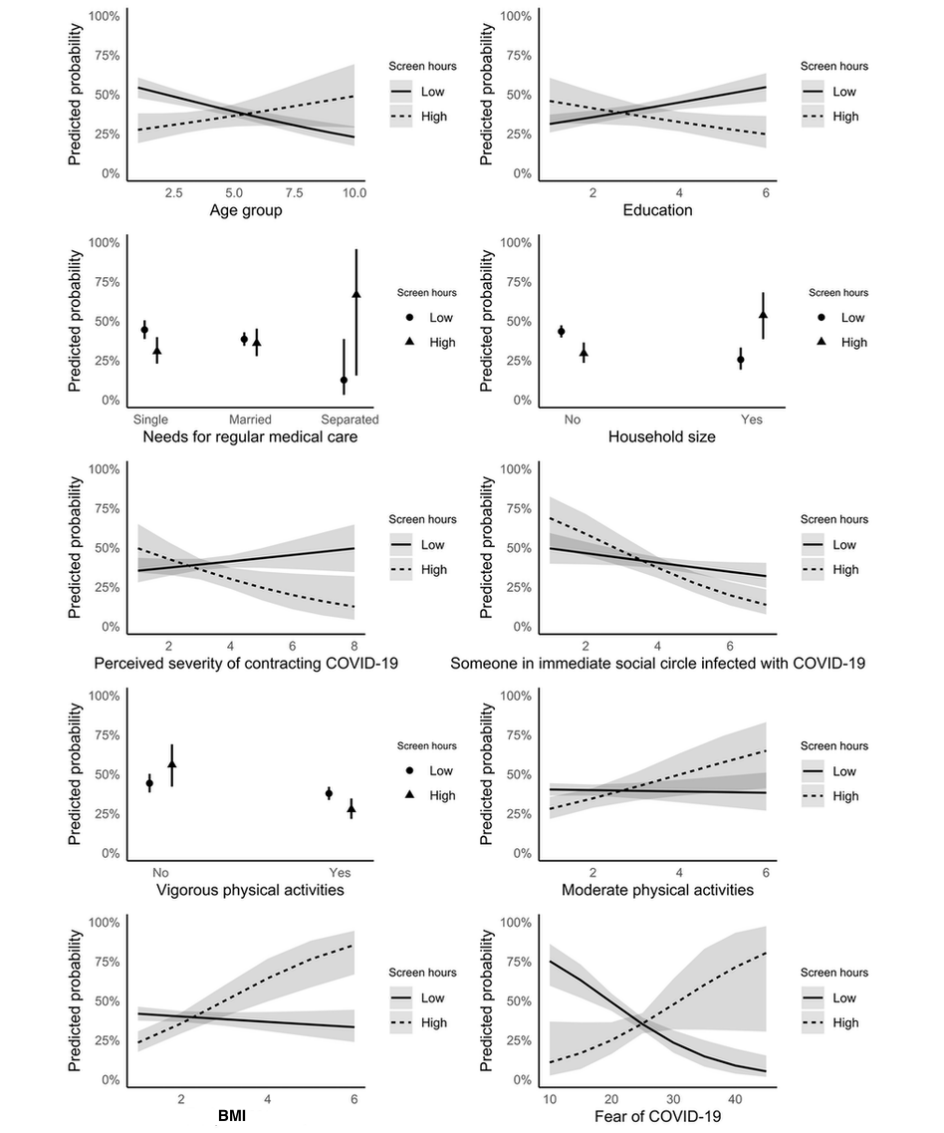
Visualizing the moderation effect of screen hour level (n=1014).

Screen hour levels moderated the association between willingness to sustain digital work and 10 variables (Table 3 and Figure 1): age, education, needs for regular medical care, household size, perceived COVID-19 severity, COVID-19 cases in the immediate social circle, and the frequency of both vigorous and moderate physical activities, along with BMI and fear of COVID-19 (*P* value for interaction <.05 for all). In the high screen time group, age (OR 1.32, 95% CI 1.18-1.49), needs for regular medical care (OR 6.48, 95% CI 3.28-13.40), BMI (OR 1.16, 95% CI 1.03-1.33), and fear of COVID-19 (OR 1.08, 95% CI 1.03-1.12) positively influenced willingness (*P*<.001 for all). Conversely, the low screen time group showed reduced willingness for these factors—age (OR 0.87, 95% CI 0.83-0.91), regular medical care (OR 0.47, 95% CI 0.33-0.65), BMI (OR 0.87, 95% CI 0.82-0.92), and fear of COVID-19 (OR 0.96, 95% CI 0.94-0.98; *P*<.001 for all). In addition, education (OR 1.30, 95% CI 1.16-1.47) and household size (OR 1.48, 95% CI 1.29-1.69) enhanced willingness in the low screen time group but negatively affected the high screen time group (education: OR 0.62, 95% CI 0.50-0.76; household size: OR 0.56, 95% CI 0.43-0.71; *P*<.001 for all). Moreover, perceived COVID-19 severity (OR 0.73, 95% CI 0.59-0.91) and presence of COVID-19 in the immediate social circle (OR 0.15, 95% CI 0.07-0.29) negatively impacted willingness in the high screen hour group, whereas the frequency of vigorous (OR 1.34, 95% CI 1.03-1.76) and moderate physical activities (OR 1.72, 95% CI 1.30-2.33) positively influenced willingness only in the high screen hour group (all *P*<.05).

**Table 3:** Moderating effects of screen hour level (n=1014)^a^.

Variable moderated by screen hour level	Low level of screen hour (≤8 h/d; n=783)			High level of screen hour (>8 h/d; n=231)			*P* values for interaction
	OR^b^ (95% CI)	*P* value	OR (95% CI)		*P* value		
Age (years)	0.87 (0.83-0.91)	<.001	1.32 (1.18-1.49)		<.001	.006	
Education level	1.30 (1.16-1.47)	<.001	0.62 (0.50-0.76)		<.001	.009	
Needs of regular medical care (yes vs no)	0.47 (0.33-0.65)	<.001	6.48 (3.28-13.40)		<.001	<.001	
Household size	1.48 (1.29-1.69)	<.001	0.56 (0.43-0.71)		<.001	.03	
Perceived severity of contracting COVID-19	0.96 (0.86-1.08)	.49	0.73 (0.59-0.91)		.005	.02	
Someone in your immediate social circle infected with COVID-19 (yes vs no)	0.94 (0.70-1.27)	.69	0.15 (0.07-0.29)		<.001	.007	
Frequency of vigorous physical activities	1.04 (0.91-1.18)	.56	1.34 (1.03-1.76)		.03	.008	
Frequency of moderate physical activities	0.96 (0.85-1.08)	.53	1.72 (1.30-2.33)		<.001	<.001	
BMI	0.87 (0.82-0.92)	<.001	1.16 (1.03-1.33)		.02	.01	
Fear of COVID-19	0.96 (0.94-0.98)	<.001	1.08 (1.03-1.12)		<.001	.02	

^a^All estimated effects were adjusted by variables that correspond to their respective phase, as listed in Table 2.

^b^OR: odds ratio.

## Discussion

### Principal Findings

This study is the first to evaluate the general population’s willingness to sustain pandemic-induced digital work, associated factors, and the moderating effect of screen hour levels. A willingness rate of 38.6% (391/1014) highlights the need for improved tele-based infrastructure and health outcome monitoring. In addition, factors positively linked to higher willingness included being employees, health professionals, having longer screen hours, and higher depression levels. Conversely, negative factors were older age, extroversion, high health literacy, perceived greater susceptibility to COVID-19, perceived community COVID-19 severity, having infected individuals in the immediate social circle, higher BMI, increased feelings of losing control, and fear of COVID-19. Moreover, screen hour levels moderated the impact of 10 factors: age, education, needs for regular medical care, household size, perceived COVID-19 severity, COVID-19 cases in the immediate social circle, and the frequency of both vigorous and moderate physical activities, along with BMI and fear of COVID-19. These findings enhance our understanding of the drivers behind digital work engagement, facilitating the customization of digital work policies and regulations, and guiding the development of strategies tailored to people with various screen hour levels.

### Considerable Level of Willingness to Sustain

Compared with a previous study in which 63.4% of energy sector employees in the United States preferred not returning to physical workplaces [8], only 38.6% of the general population in Hong Kong expressed willingness to continue digital work. This difference likely arises from the energy sector’s compatibility with remote work. To meet the universal demand for better digital support, organizations should overcome challenges, such as inadequate infrastructure and training, and inflexibility in physical roles. In addition, there is a critical need to monitor and prevent adverse health outcomes associated with digital work, such as sedentary behavior, musculoskeletal disorders, eye strain, and mental health issues. Key strategies should include investing in technology, fostering a culture of flexible work, enhancing digital literacy, revising policies to ensure equitable access to remote work, and establishing recommendations and standards for digital workplace health.

### Associated Factors of Willingness to Sustain Digital Work Mode

Understanding factors associated with sustaining digital work is key to leveraging this shift. Consistent with prior studies [24,25], older adults and extroverts are less inclined toward digital work due to technological discomfort and reduced social interactions, respectively. Tailored training for older adults and enhanced digital networking can address these issues. The other 11 associated factors are newly identified.

Specifically, for negative factors, individuals with high health literacy might recognize the health risks of prolonged digital engagement, showing reduced willingness; this highlights the need for clear ergonomic guidelines and health strategies. Environmental safety concerns [26], such as increased perceptions of infection risk, community COVID-19 severity, having infected individuals in the immediate social circle, and fear of COVID-19, also lower willingness, emphasizing the need for transparent safety protocols to reassure those concerned about infection risks. Conversely, individuals with higher BMI may prefer remote work to avoid commuting and stigma associated with social interactions in physical workplaces [27], underscoring the need for inclusive and ergonomic remote work policies. In addition, those feeling a loss of control find remote environments isolating and unpredictable without the structured support of traditional workplaces [28], underlining the importance of structured routines and clear digital tool training to instill organization and control, thereby increasing the appeal of digital work.

In contrast, for positive factors, employees often exhibit greater willingness, drawn by perceived benefits such as improved work-life balance and efficiency [8]. Organizations should therefore promote flexibility and efficiency, tailoring practices based on employee feedback. Health professionals, more adept with digital tools and telehealth [29], also show a higher propensity to sustain digital modes. Continued investment in digital health care platforms and comprehensive training to keep up with technological advancements are beneficial. Similarly, those accustomed to long screen hours likely find digital environments more conducive to productivity and work-life balance. However, this finding underscores the importance of focusing on ergonomic solutions and wellness programs to mitigate health risks associated with sedentary behavior. Individuals with depression may prefer digital work, as their typical avoidance symptoms might make traditional settings more stressful [30], suggesting that digital methods could enhance mental support, therapy, and access to digital counseling, fostering a supportive work culture.

### Moderating Effects of Screen Hour Level

The moderating effect of screen hour levels, indicating different degrees of digital engagement in work, highlights the diverse mechanisms affecting willingness to sustain digital work across groups. First, older age, regular medical follow-ups, higher BMI, and greater COVID-19 fear have opposite impacts in high versus low screen time groups. Older individuals with longer screen hours typically possess digital work skill and recognize its benefits. Individuals with high screen hours likely integrated medical follow-ups with their digital work before the pandemic, while those with fewer screen hours struggled with coordination, leading to contrasting moderation effects. Individuals with high BMI in the low screen hour group may fear increased sedentary behavior worsening their condition and facing social stigma [31], while those in the high screen hour group see digital work as a way to avoid such stigma [27]. Similarly, fear of COVID-19 varies; high screen time users see continued digital work as protective, whereas those with less screen time, encountering less alarming news on websites [32], feel safer pursuing outdoor activities.

Previously, higher education was considered a positive factor [33] and household size a negative one [34]. However, in the high screen hour group, both higher education and larger households detract from digital work appeal, likely due to increased health risk awareness of digital work and more disruptions, respectively. Conversely, in the low screen hour group, higher education and larger households positively impact digital work by enhancing the ability to manage digital challenges and improving work-life balance.

Finally, infection concerns and COVID-19 exposure negatively impact only the high screen time group, possibly increasing stress and reducing productivity [35]. Conversely, frequent moderate and vigorous physical activities benefit this group by potentially offsetting sedentariness and managing stress [36].

The moderating effects of screen time exposure call for a customized approach that considers varying digital demands to enhance digital work acceptance. For high screen hour group, considering regular medical follow-ups significantly increase willingness to engage in digital work, strategies to incorporate chronic disease management into tele-based work through digital platforms, along with mitigating health impacts from prolonged digital engagement via health education and less disruptive environments such as immersive setups [37], are beneficial. Policies promoting an active lifestyle with incentives for physical activities [38] can greatly enhance willingness to sustain digital modes. For low screen hour group, boosting digital literacy with targeted training and improving digital tool access can positively affect perceptions of digital work benefits. In addition, addressing the reluctance to sustain digital work in this group during social distancing for future pandemics is crucial for public health planning.

### Limitations

Several limitations are worth noting. First, the study included participants only from Hong Kong, which may limit the generalizability of the findings to other countries or cultures. Second, the data collection relied on a digital platform, potentially excluding individuals with low socioeconomic status, limited digital literacy, or inadequate access to the internet or digital devices, or including more participants proficient in digital tools. Such selection bias might potentially inflate prevalence rates and skewing the factors perceived to influence willingness to sustain digital work. Future studies should adopt varied data-gathering techniques to enhance sample representativeness. Third, our study’s cross-sectional design constrains insights into how individuals’ willingness to sustain digital work may shift over time as the pandemic evolves and limits our ability to infer causality. Longitudinal or experimental studies are needed for a more thorough understanding. Fourth, in the phase 2 regression, we included 19 independent variables without correcting for type I error, necessitating cautious interpretation of small-effect associations. Future research should rigorously control for type I error to establish causality more reliably. Finally, due to the lack of established tools to measure willingness to sustain pandemic-induced digital work, we used a single question, potentially oversimplifying the assessment. Future research should develop validated tools for more accurate evaluations.

### Conclusions

Our study shows that 38.6% (391/1014) of the general population in Hong Kong who increased their engagement in digital work during the pandemic are willing to continue this mode, highlighting the significant impact of the pandemic on digital work adoption and its potential sustainability. These findings emphasize the need for improved telework infrastructure and training; vigilant health surveillance to prevent issues such as musculoskeletal disorders, eye strain, and sedentary behavior; and the establishment of comprehensive digital work health guidelines and workplace standards. The high adoption rate also indicates promising prospects for expanding telehealth services within these populations. Furthermore, we identified 13 factors influencing this willingness, of which 11 were newly identified, and found that screen time levels moderate the impact of another 10 factors. These findings enhance our understanding of the drivers behind digital work engagement, enabling the customization of digital work policies, settings, and regulations. The moderation effects of screen time offer a detailed perspective on how these factors differ across various screen hour–demanding individuals, supporting the development of more targeted strategies to enhance their digital work acceptance where needed.

## Appendix

Multimedia Appendix 1 Web-based questionnaire.

Multimedia Appendix 2 Structured multiphase logistic regression analysis of willingness to sustain digital work, with statistics for the adjusting variables (n=1014).

## Abbreviations

CHERRIES: Checklist for Reporting Results of Internet E-Surveys
OR: odds ratio
