# Supplementary material for: Prevalence and Factors Associated With Willingness to Sustain Pandemic-Induced Digital Work in the General Population and Moderating Effects of Screen Hours: Cross-Sectional Study
Source: J Med Internet Res. 2024 May 28;26:e53321. doi: 10.2196/53321 (PMC11167320; doi:10.2196/53321)
Supplement: Multimedia Appendix 1 [file jmir_v26i1e53321_app1.docx]

**A. Personal Information**

1. What is your age?

1. 18-24
2. 25-29
3. 30-34
4. 35-39
5. 40-44
6. 45-49
7. 50-54
8. 55-59
9. 60-64
10. 65 or older

2. What is your current marital status?

1. Single
2. Married/Cohabitation/common-law
3. Separated/divorced/widowed

3. What is your gender?

1. Male
2. Female

4. What is the highest level of education you have ever completed?

1. Primary school or below
2. Secondary school or equivalent
3. College/certificate/technical diploma
4. Associate degree
5. Bachelor’s degree
6. Graduate degree or more (e.g. Master’s, Doctorate, MD)

5. What is your current occupation?

1. Student
2. Employee
3. Laid off
4. Job seeking and unemployed
5. Not in the workforce (e.g. Homemaker, unemployed, not looking for work)
6. Retired
7. Self-employed

6. What is your perceived social rank? (1-5, 1=lowest, 5=highest)

1. Might not have a job/go to the worst schools, don’t have enough money, don’t live in a nice place


5. Have the best jobs/go to the best schools, have lots of money, live in nice places

7. What is your height? (cm) (1 foot = 30.5 cm; 1 inch = 2.54 cm)

______ cm

8. What is your weight? (kg) (1 pound = 0.45 kg)

______ kg

9. Were you on regular medical follow-up before COVID-19?

1. Yes
2. No

10. Are you a practicing health professional? (e.g. nurse, medical doctor, pharmacist, etc.)

1. Yes
2. No

11. How many children less than 18 years old do you have? _________ (no children = 0)

12. How many people live with you (including yourself)? _________

13. What is the estimated size (saleable/net area) of where you are living? (1 ft^2^ = 0.1 m^2^) ________ m^2^

**B. COVID-19 related-knowledge**

| 14. | How would you rate your knowledge level on COVID-19? | Very poor | Poor | Slight poor | Neither poor nor good | Slightly good | Good | Very good |
| --- | --- | --- | --- | --- | --- | --- | --- | --- |
| 15. | How would you rate your knowledge level on how to prevent spread of COVID-19? | Very poor | Poor | Slight poor | Neither poor nor good | Slightly good | Good | Very good |
| 16. | Do you think you have adequate knowledge about COVID-19? | Absolutely inadequate | Moderately inadequate | Slightly inadequate | Neither adequate not inadequate | Slightly adequate | Moderately adequate | Absolutely adequate |
| 17. | During the pandemic, how susceptible do you consider yourself to an infection with COVID-19? | Not susceptible | Moderately not susceptible | Slightly not susceptible | Neutral | Slightly susceptible | Moderately susceptible | Very susceptible |
| 18. | During the pandemic, how severe would contracting COVID-19 be for you? | Not severe | Moderately not severe | Slightly not severe | Neutral | Slightly severe | Moderately severe | Very severe |
| 19. | During the pandemic, how severe is the spread COVID-19 in your community? | Not severe | Moderately not severe | Slightly not severe | Neutral | Slightly severe | Moderately severe | Very severe |

**C. COVID-19 Status**

20. Are you or have you been infected with COVID-19?

1. Yes, confirmed
2. No

21. Do you know people in your immediate social environment who are or have been infected with COVID-19?

1. Yes, confirmed
2. No

**Validation question**

22. Where does the sun rise every day?

1. West
2. North
3. South
4. East

**D. Lifestyles**

Questions 23-28 are related to your lifestyle during the COVID-19 pandemic.

23. Do you drink alcohol?

1. Never
2. Quit
3. Occasionally, only on special occasions
4. Less than once per month
5. 1-3 times per month
6. 1-3 times per week
7. 4-6 times per week
8. Everyday

24. Do you smoke tobacco? (1 cigarette counted once)

1. Never
2. Quit
3. Occasionally, only on special occasions
4. Less than once per month
5. 1-3 times per month
6. 1-3 times per week
7. 4-6 times per week
8. Everyday

25. In the past week, what is your typical screen time on electronic devices per day? (Including watching TV, playing video games, using laptop computers, smart phones, and/or tablets)

______ hours

26. In the past week, how many days did you do vigorous physical activities for at least 10 minutes at a time? Vigorous physical activities are those that make you breathe much harder than normal (e.g. running, aerobics, football, swimming, heavy physical work, jogging, etc.).

1. None
2. One day
3. Two days
4. Three days
5. Four days
6. Five days or more

27. In the past week, how many days did you do moderate physical activities for at least 10 minutes at a time? Moderate physical activities are those that make you breathe somewhat harder than normal (e.g. bicycling, washing cars, fast walking, cleaning windows, etc.).

1. None
2. One day
3. Two days
4. Three days
5. Four days
6. Five days or more

28. In the past week, how much time did you spend sitting per day, on average? (Including sitting at home, in an office, in front of the computer, watching TV, reading, visiting friends, in a car, etc.)

_______ hours per day

**E. Fear Scale**

The following questions are about how you feel when you think about the possibility of getting COVID-19. Different people have different feelings. Please choose one answer for each statement below.

|  | Strongly Disagree | Disagree | Neutral | Agree | Strongly Agree |
| --- | --- | --- | --- | --- | --- |
| 1. The thought of COVID-19 scares me. |  |  |  |  |  |
| 1. When I think about COVID-19, I feel nervous. |  |  |  |  |  |
| 1. When I think about COVID-19, I get upset. |  |  |  |  |  |
| 1. When I think about COVID-19, I get depressed |  |  |  |  |  |
| 1. When I think about COVID-19, I get jittery. |  |  |  |  |  |
| 1. When I think about COVID-19, my heart beats faster. |  |  |  |  |  |
| 1. When I think about COVID-19, I feel uneasy. |  |  |  |  |  |
| 1. When I think about COVID-19, I feel anxious. |  |  |  |  |  |

**F. eHealth Literacy (eHEALs)**

According to your most recent Internet search for information on COVID-19, to what extent do the following statements reflect your situation?

|  | Strongly  disagree | Disagree | Undecided | Agree | Strongly  agree |
| --- | --- | --- | --- | --- | --- |
| 1. I know how to find helpful health resources on the Internet |  |  |  |  |  |
| 1. I know how to use the Internet to answer my questions about health |  |  |  |  |  |
| 1. I know what health resources are available on the Internet |  |  |  |  |  |
| 1. I know where to find helpful health resources on the Internet |  |  |  |  |  |
| 1. I know how to use the health information I find on the Internet to help me |  |  |  |  |  |
| 1. I have the skills I need to evaluate the health resources I find on the Internet |  |  |  |  |  |
| 1. I can tell high quality health resources from low quality health resources on the Internet |  |  |  |  |  |
| 1. I feel confident in using information from the Internet to make health decisions |  |  |  |  |  |

**G. PHQ-4**

Over the last two weeks, how often have you been bothered by the following problems?

|  | Not at all | Several days | More than half the days | Nearly every day |
| --- | --- | --- | --- | --- |
| 1. Feeling nervous, anxious or on edge |  |  |  |  |
| 1. Not being able to stop or control worrying |  |  |  |  |
| 1. Feeling down, depressed or hopeless |  |  |  |  |
| 1. Little interest or pleasure in doing things |  |  |  |  |

**H. Out-of-Control Scale**

For each of the following items related to the current COVID-19 pandemic, please indicate your level of agreement. The available options are: strongly disagree, disagree, somewhat disagree, somewhat agree, agree, and strongly agree.

*Note: The validation paper for the "Out-of-Control Scale" has not yet been published. As a result, we have chosen to withhold the specific questions from this supplementary file to maintain confidentiality.*

1. **Perceived personality**

Do you primarily identify as an introvert or an extrovert?

a) Introverted (I like to do things alone or with one or two very close friends, I tend to be quiet and restrained)

B) Outgoing (I am sociable, enjoy lively gatherings and meeting new people)

1. **Changes in work mode due to the COVID-19 pandemic and willingness to sustain**
2. Compared to the period before the COVID-19 pandemic, have you experienced the following changes in your work or learning mode?

More online working or learning: a) Yes b) No

No change in my work or learning mode: a) Yes b) No

1. Do you willing to sustain such pandemic-induced digital work mode?
2. yes

b) no
