# Supplementary material for: Prevalence and Factors Associated With Willingness to Sustain Pandemic-Induced Digital Work in the General Population and Moderating Effects of Screen Hours: Cross-Sectional Study
Source: J Med Internet Res. 2024 May 28;26:e53321. doi: 10.2196/53321 (PMC11167320; doi:10.2196/53321)
Supplement: Multimedia Appendix 2 [file jmir_v26i1e53321_app2.docx]

| **Variables** | | | | ***OR*** | **95% CI** | ***p*** | **VIF** |
| --- | --- | --- | --- | --- | --- | --- | --- |
| **Phase 1** | | | | | | | |
| Age (1-10)^a^ | | | | 0.87 | (0.81, 0.94) | < .001 | 1.35 |
| Educational level (1-6)^b^ | | | | 0.98 | (0.88, 1.10) | .768 | 1.16 |
| **Marital status (ref: single)** | | | | | | | 1.17 |
|  | Married/Cohabitation/Common-law | | | 1.24 | (0.87, 1.78) | .238 |  |
|  | Separated/Divorced/Widowed | | | 0.61 | (0.16, 1.88) | .422 |  |
| **Gender (ref: female)** | | | | | | | 1.03 |
|  | Male | | | 0.79 | (0.61, 1.04) | .092 |  |
| **Occupational status (ref: student)** | | | | | | | 1.04 |
|  | Self-employed | | | 1.48 | (0.47, 4.42) | .485 |  |
|  | Employee | | | 3.12 | (1.59, 6.45) | .001 |  |
| Needs for regular medical care (Yes vs No) | | | | 0.83 | (0.57, 1.18) | .298 | 1.05 |
| Practicing health professional (Yes vs No) | | | | 3.32 | (1.49, 7.82) | .004 | 1.02 |
| Number of children less than 18 years old | | | | 0.87 | (0.69, 1.10) | .258 | 1.24 |
| Number of people in the household | | | | 1.00 | (0.87, 1.15) | .999 | 1.17 |
| House size, m^2^ | | | | 1.00 | (0.99, 1.01) | .458 | 1.13 |
| Perceived social rank | | | | 1.02 | (0.83, 1.25) | .846 | 1.08 |
| Personality (Extroverts vs Introverts) | | | | 0.66 | (0.51, 0.86) | .002 | 1.01 |
| **Phase 2** | | | | | | | |
| *Age (1-10)^a^* | | | | *0.88* | *(0.82, 0.93)* | *< .001* | *1.12* |
| ***Occupational status (ref: student)*** | | | |  |  |  | *1.04* |
|  | *Self-employed* | | | *1.62* | *(0.49, 5.08)* | *.418* |  |
|  | *Employee* | | | *3.49* | *(1.75, 7.34)* | *.001* |  |
| *Practicing health professional (Yes vs No)* | | | | *4.17* | *(1.82, 10.04)* | *.001* | *1.02* |
| *Personality (Extroverts vs Introverts)* | | | | *0.72* | *(0.54, 0.96)* | *.025* | *1.04* |
| E-health literacy | | | | 0.96 | (0.93, 0.98) | < .001 | 1.08 |
| Knowledge on COVID-19 | | | | 0.84 | (0.68, 1.04) | .120 | 1.39 |
| Knowledge on preventing the spread of COVID-19 | | | | 1.11 | (0.90, 1.37) | .352 | 1.44 |
| Perceived adequacy of knowledge about COVID-19 | | | | 0.90 | (0.73, 1.09) | .272 | 1.34 |
| Perceived susceptibility to an infection with COVID-19 | | | | 0.84 | (0.74, 0.96) | .009 | 1.17 |
| Perceived severity of contracting COVID-19 | | | | 1.08 | (0.93, 1.25) | .315 | 1.31 |
| Severity of the spread COVID-19 in your community | | | | 0.73 | (0.63, 0.84) | < .001 | 1.30 |
| Ever infected with COVID-19 (Yes vs No) | | | | 1.03 | (0.74, 1.42) | .876 | 1.14 |
| Someone in your immediate social circle infected with COVID-19 (Yes vs No) | | | | 0.64 | (0.46, 0.88) | .006 | 1.12 |
| Frequency of alcohol drinking per week | | | | 1.01 | (0.92, 1.11) | .815 | 1.13 |
| Frequency of smoking per week | | | | 0.97 | (0.92, 1.03) | .360 | 1.11 |
| Screen hours | | | | 1.09 | (1.03, 1.15) | .002 | 1.37 |
| Sitting hours | | | | 0.97 | (0.92, 1.02) | .276 | 1.35 |
| Frequency of vigorous physical activities per week | | | | 0.95 | (0.83, 1.08) | .410 | 1.18 |
| Frequency of moderate physical activities per week | | | | 1.10 | (0.97, 1.24) | .146 | 1.18 |
| **Phase 3** | | | | | | | |
| *Age (1-10)^a^* | | | | *0.91* | *(0.85, 0.97)* | *.002* | *1.11* |
| ***Occupational status (ref: student)*** | | | |  |  |  | *1.03* |
|  | | *Self-employed* | | *1.60* | *(0.49, 4.93)* | *.417* |  |
|  | | *Employee* | | *3.59* | *(1.82, 7.50)* | *< .001* |  |
| *Practicing health professional (Yes vs No)* | | | | *3.44* | *(1.55, 8.05)* | *.003* | *1.00* |
| *Personality (Extroverts vs Introverts)* | | | | *0.71* | *(0.54, 0.93)* | *.014* | *1.01* |
| *Perceived susceptibility to an infection with COVID-19* | | | | *0.85* | *(0.76, 0.96)* | *.010* | *1.10* |
| *Severity of the spread COVID-19 in your community* | | | | *0.73* | *(0.64, 0.82)* | *< .001* | *1.10* |
| *Someone in your immediate social circle infected with COVID-19 (Yes vs No)* | | | | *0.63* | *(0.47, 0.84)* | *.002* | *1.02* |
| *Screen hours* | | | | *1.07* | *(1.03, 1.12)* | *.001* | *1.07* |
| Body mass index | | | | 0.94 | (0.90, 0.99) | .023 | 1.03 |
| **Phase 4** | | | | | | | |
| *Age (1-10)^a^* | | | | *0.92* | *(0.86, 0.98)* | *.008* | *1.11* |
| ***Occupational status (ref: student)*** | | | |  |  |  | *1.04* |
|  | | | *Self-employed* | *1.48* | *(0.45, 4.56)* | *.504* |  |
|  | | | *Employee* | *3.38* | *(1.69, 7.15)* | *.001* |  |
| *Practicing health professional (Yes vs No)* | | | | *3.34* | *(1.46, 8.04)* | *.005* | *1.01* |
| *Personality (Extroverts vs Introverts)* | | | | *0.74* | *(0.56, 0.98)* | *.033* | *1.01* |
| *Perceived susceptibility to an infection with COVID-19* | | | | *0.87* | *(0.77, 0.98)* | *.026* | *1.12* |
| *Severity of the spread COVID-19 in your community* | | | | *0.74* | *(0.65, 0.83)* | *< .001* | *1.12* |
| *Someone in your immediate social circle infected with COVID-19 (Yes vs No)* | | | | *0.60* | *(0.44, 0.81)* | *.001* | *1.03* |
| *Screen hours* | | | | *1.07* | *(1.03, 1.12)* | *.001* | *1.07* |
| *Body mass index* | | | | *0.94* | *(0.89, 0.99)* | *.025* | *1.06* |
| Out of control | | | | 0.96 | (0.93, 0.98) | .002 | 1.22 |
| Fear of COVID-19 | | | | 0.96 | (0.94, 0.98) | .001 | 1.32 |
| Anxiety | | | | 1.07 | (0.90, 1.28) | .437 | 1.52 |
| Depression | | | | 1.20 | (1.01, 1.44) | .039 | 1.47 |

^a^Age group: 1 to 10 corresponding to 18-24, 25-29, 30-34, 35-39, 40-44, 45-49, 50-54, 55-59, 60-64, and older than 65-year-old, respectively.

^b^Educational level: 1 to 6 corresponding to primary or below, secondary, college, associated degree, bachelor, and graduate, respectively.

The adjusting variables in Phases 2, 3, and 4 were marked in italic.
